# Comparative analysis of full-length transcriptomes based on hybrid population reveals regulatory mechanisms of anthocyanin biosynthesis in sweet potato (*Ipomoea batatas* (L.) Lam)

**DOI:** 10.1186/s12870-020-02513-1

**Published:** 2020-06-29

**Authors:** Zhen Qin, Fuyun Hou, Aixian Li, Shuxu Dong, Chengxing Huang, Qingmei Wang, Liming Zhang

**Affiliations:** 1 Crop Research Institute, Shandong Academy of Agricultural Sciences, No. 202 Industry North Road, Jinan City, 250100 Shandong Province China; 2 Scientific Observing and Experimental Station of Tuber and Root Crops in Huang-Huai-Hai Region, Ministry of Agriculture, Jinan Shandong, China; 3 Jining Academy of Agricultural Sciences, Jining, Shandong China

**Keywords:** Sweet potato, Full-length transcriptome, Anthocyanin biosynthesis, Transcription factor, Weighted gene co-expression network analysis

## Abstract

**Background:**

Sweet potato (*Ipomoea batatas* (L*.*) Lam.) is a highly heterozygous autohexaploid crop with high yield and high anthocyanin content. Purple sweet potato is the main source of anthocyanins, and the mechanism of anthocyanin biosynthesis in storage roots has not been fully revealed.

**Results:**

In order to reveal the mechanism of anthocyanin biosynthesis and identify new homologous genes involved in anthocyanin biosynthesis in the storage roots of sweet potato, we used Ningzishu 1 and Jizishu 2 as parents to construct a F_1_ hybrid population. Seven anthocyanin-containing lines and three anthocyanin-free lines were selected for full-length and second-generation transcriptome analyses. A total of 598,375 circular consensus sequencing reads were identified from full-length transcriptome sequencing. After analysis and correction of second-generation transcriptome data, 41,356 transcripts and 18,176 unigenes were obtained. Through a comparative analysis between anthocyanin-containing and anthocyanin-free groups 2329 unigenes were found to be significantly differentially expressed, of which 1235 were significantly up-regulated and 1094 were significantly down-regulated. GO enrichment analysis showed that the differentially expressed unigenes were significantly enriched in molecular function and biological process. KEGG enrichment analysis showed that the up-regulated unigenes were significantly enriched in the flavonoid biosynthesis and phenylpropanoid biosynthesis pathways, and the down-regulated unigenes were significantly enriched in the plant hormone signal transduction pathway. Weighted gene co-expression network analysis of differentially expressed unigenes revealed that anthocyanin biosynthesis genes were co-expressed with transcription factors such as MYB, bHLH and WRKY at the transcription level.

**Conclusions:**

Our study will shed light on the regulatory mechanism of anthocyanin biosynthesis in sweet potato storage roots at the transcriptome level.

## Background

Sweet potato is a highly heterozygous hexaploid and an important food crop, which has been grown around the world with an area of 9.2 million hectares (FAO, 2017). Sweet potato has the characteristics of high yield, barren tolerance and wide applicability [1]. Purple sweet potatoes contain anthocyanins. Anthocyanins are the products of the secondary metabolic pathway of plant flavonoids and have been applied as natural water-soluble pigments [2]. Studies have shown that anthocyanins have antioxidant activities and play a preventive role in many human diseases [3]. Anthocyanins in purple sweet potato have high light and thermal stabilities [4], which make it an excellent raw material for extracting anthocyanins.

The mechanism of plant anthocyanin biosynthesis has been extensively studied [5, 6]. Anthocyanin biosynthesis, using phenylalanine as the substrate, employs the following enzymes, phenylalanine ammonialyase (PAL), cinnamate4-hydroxylase (C4H), cinnamate-4-hydroxylase (C4L), chalcone synthase (CHS), chalcone isomerase (CHI), flavanone 3-hydroxylase (F3H), flavonoid 3′-hydroxylase (F3′H), flavonoid-3′5′-hydroxylase (F3′5′H), dihydroflavonol reductase (DFR), leucoanthocyanidin dioxygenase (LDOX), anthocyanidin synthase (ANS), and UDP-glucose flavonoid 3-o-glycosyltransferase (UF3GT) [7]. The genes encoding these enzymes are mainly transcriptionally regulated by the MYB-bHLH-WDR (MBW) complex, which consists of MYB, bHLH and WD40 [8]. Transcription factors involved in the regulation of anthocyanin biosynthesis genes also include WRKY [9], bZIP [10], and NAC [11], etc. Studies have shown that miRNAs are also involved in the regulation of plant anthocyanin biosynthesis [12, 13]. Transcription factors that regulate the expression of structural genes in anthocyanin biosynthesis are differentially regulated by biological and environmental factors such as hormones, sugars, and light [14]. These studies indicate that plant anthocyanin biosynthesis is regulated by multiple factors.

Three studies have been constructed on transcriptome analyses of anthocyanins of sweet potato storage roots. Xie [15] compared two RNA-sequence datasets of Jingshu 6 and Guangshu 87 and found that UDP-glucose-flavonoid 3-*O*-glucosyltransferase is one of the key enzymes in the anthocyanin biosynthesis pathway. Ma [16] and Zhao [17] discovered many structural genes and transcription factors related to anthocyanin biosynthesis through transcriptome analyses of sweet potato mutants.

In this study, we selected Ningzishu 1 with low anthocyanin content as the female parent and Jizishu 2 with high anthocyanin content as the male parent to construct F_1_ hybrid populations. Seven anthocyanin-containing lines and three anthocyanin-free lines were selected from the hybrid population for transcriptome sequencing. By comparative analyses of the full-length transcriptome data of the hybrid population, this study was aimed at revealing the mechanism of anthocyanin biosynthesis and identifying new homologous genes involved in anthocyanin biosynthesis in the storage roots of sweet potato.

## Results

### Determination of anthocyanin content

**Fig. 1:**
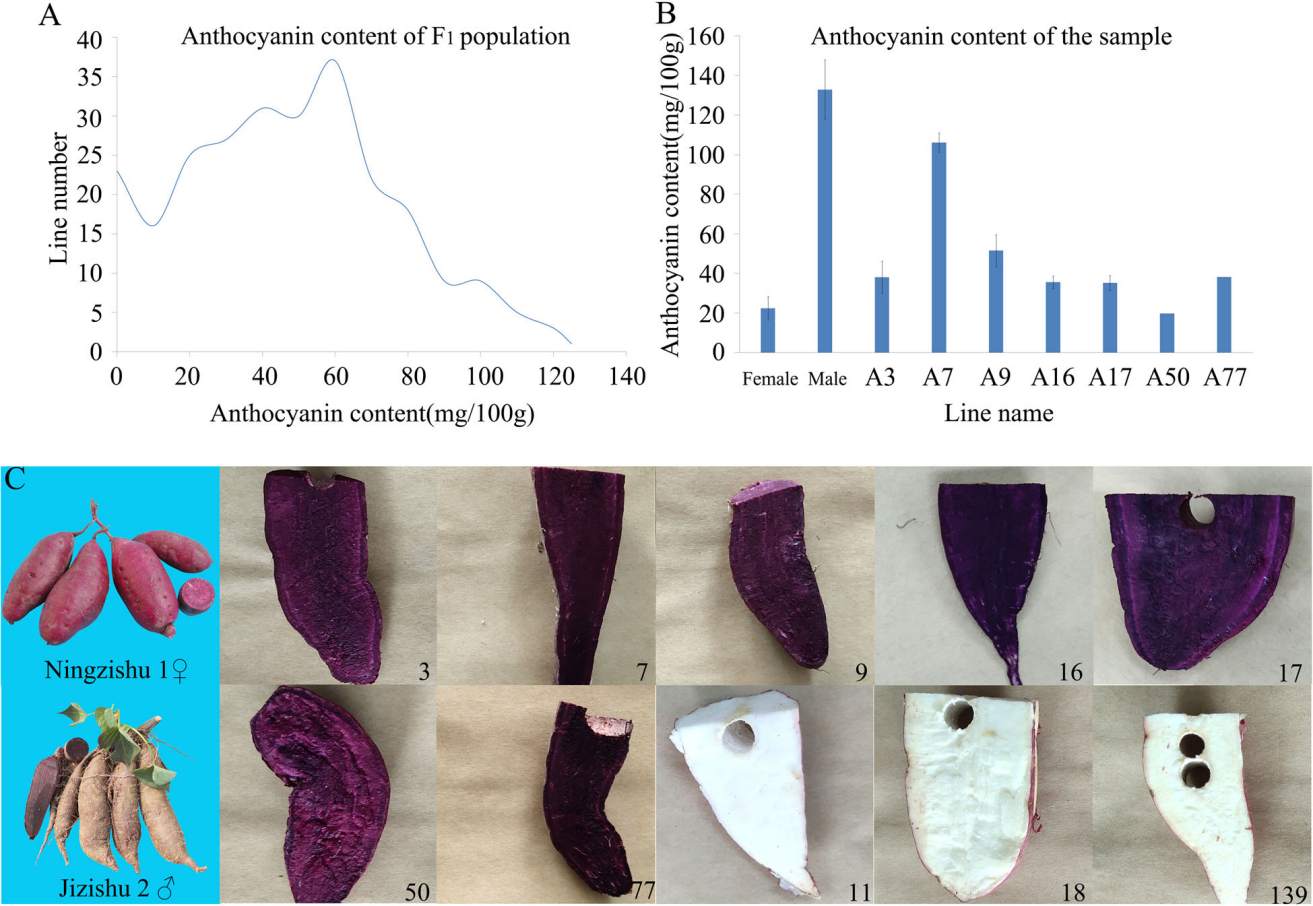
Anthocyanin content in sweet potato hybrid populations. A. Anthocyanin content in 256 lines of sweet potato hybrid populations. The X-axis represents the anthocyanin content, and the Y-axis represents the number of lines. B. Anthocyanin content of seven anthocyanin-containing lines for transcriptome sequencing and two parents. The X-axis represents the line name, and the Y-axis represents the anthocyanin content. C. Photographs of ten lines used for transcriptome sequencing and two parents

We collected the population lines from two locations each year for three consecutive years and determined the anthocyanin content (Fig. 1a). Among the F_1_ population, the line with the highest anthocyanin content was 125 mg/100 g fresh weight, and some of the lines did not contain anthocyanin.

### Full-length mRNA sequencing of sweet potato storage roots

**Fig. 2:**
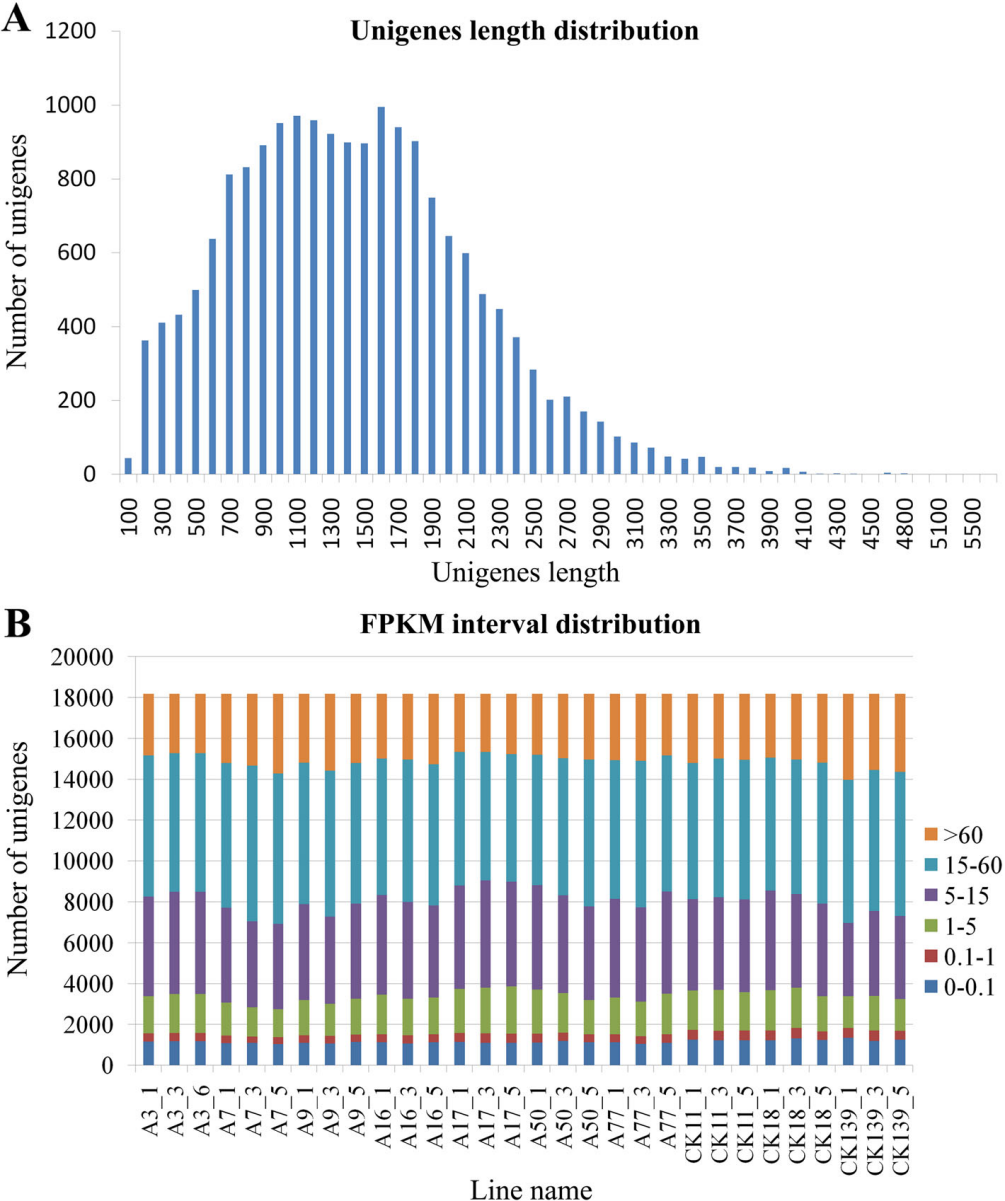
Length distribution and FPKM interval distribution of unigenes. A. Length distribution of unigenes. The X axis represents the length of unigenes, and the Y axis represents the number of unigenes. B FPKM interval distribution of unigenes. The X-axis represents sample name, and the Y-axis represents the number of unigenes

We selected seven anthocyanin-containing lines and three anthocyanin-free lines from developing storage roots of sweet potato F_1_ hybrid population (Fig. 1b and c). Nine independent individuals from each line were selected and divided equally into three samples for total RNA extraction and second-generation transcriptome sequencing. No less than 8G of clean data was obtained for each sample. At the same time, an equal amount of total RNA was mixed from each sample for third-generation transcriptome library construction (single-molecule real-time, SMRT) and sequencing analysis. A total of 598,375 circular consensus sequencing (CCS) reads ranging from 50 bp to 14,913 bp with an average length of 1234 bp were generated, including 459,960 full-length non-chimeric (FLNC) sequences and 121,847 non-full-length non-chimeric (NFL) sequences. The length of the full-length non-chimeric sequence was from 50 bp to 13,622 bp. After the sequences were corrected by second-generation transcriptome data, 41,356 transcripts were obtained, ranging in length from 54 bp to 5567 bp with an average length of 1200 bp, of which 41,027 were high-quality transcripts and 329 were low-quality transcripts. Deduplication of transcripts yielded 18,176 unigenes, ranging in length from 55 bp to 5567 bp with an average length of 1393 bp. The length distribution of the unigenes is shown in Fig. 2a. The FPKM interval distribution of the unigenes is showed in Fig. 2b. Pearson analysis of the transcriptome data found that the three replicates of each line had good consistency and met the requirements of subsequent analysis (Additional file 1: Table S1).

### Function annotation of full-length unigenes in sweet potato

We annotated the obtained unigenes based on seven databases, and the results are shown in Table 1. A total of 95.64% of the unigenes were annotated by alignment with at least one database, which provided a basis for subsequent analysis of differentially expressed genes. GO (Gene Ontology) enrichment analysis decrypts gene function by an internationally standardized classification system [18]. GO enrichment analysis found that 10,483 unigenes were significantly enriched in biological process, cellular component and molecular function. Among them, biological process was the majority of the GO terms. Within the biological process, the majority of the GO terms were assigned to metabolic process (5037 unigenes), cellular process (4925 unigenes), and single-organism process (3507 unigenes). Within the cellular component, the majority of the GO terms were assigned to cell (2305 unigenes), cell part (2305 unigenes), and organelle (1706 unigenes). Within the molecular function, the majority of the GO terms were binding (5836 unigenes) and catalytic activity (4600 unigenes) (Additional file 2: Figure S1). The KOG (euKaryotic Ortholog Groups) enrichment analysis reveals a conserved core of eukaryotic genes and diversification associated with eukaryotic genomes evolution [19]. The KOG analysis demonstrated that 10,407 unigenes were assigned to 25 functional clusters. As shown in Additional file 3: Figure S2, the four largest categories were general function prediction only (1626 unigenes), posttranslational modification, protein turnover, chaperones (1398 unigenes), signal transduction mechanisms (1010 unigenes), and translation, ribosomal structure and biogenesis (916 unigenes). KEGG (Kyoto Encyclopedia of Genes and Genomes) enrichment analysis can be used to identify functional genes and understands genes functions and interactions in biosynthetic pathways [20]. In the KEGG annotation, 16,026 unigenes were annotated in the KEGG database (Additional file 4: Figure S3). The largest pathway was the translation pathways containing 881 unigenes. The second and the third pathway were the signal transduction pathways and the carbonydrate metabolism pathways, containing 762 and 684 unigenes, respectively.

**Table 1:** Unigenes annotation information

Database	Unigene number	Percentage
NT	17,084	93.99
NR	16,274	89.54
KEGG	16,026	88.17
SwissProt	13,532	74.45
GO	10,483	57.67
Pfam	10,483	57.67
KOG	10,407	57.26
at least one Database	17,384	95.64
all Databases	7521	41.38

### Analysis of differentially expressed unigenes between anthocyanin-containing lines and anthocyanin-free lines

**Fig. 3:**
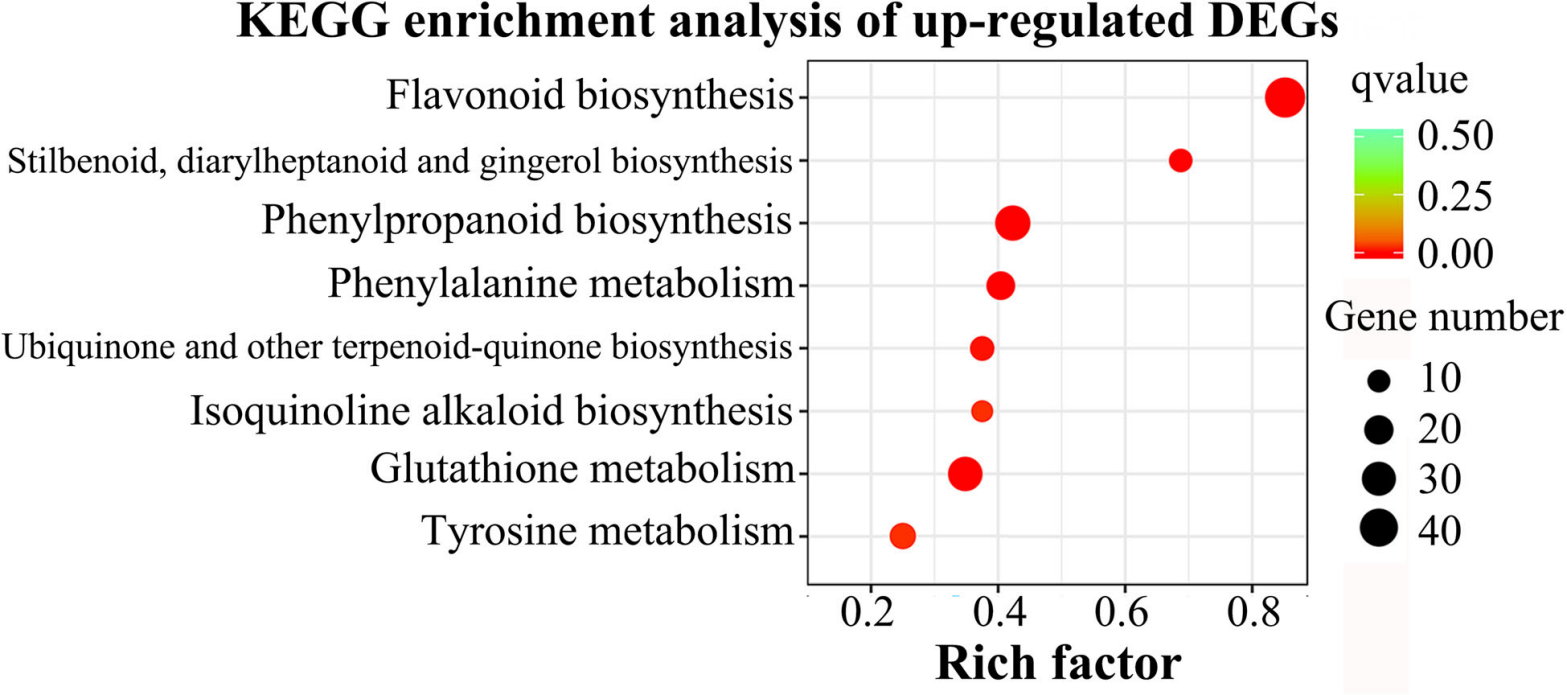
KEGG enrichment analysis of up-regulated DEGs. The X-axis represents the rich factor corresponding to the pathway, and the Y-axis represents the name of the pathway. The size of the qvalue is represented by the color of the dot. The smaller the qvalue, the closer the color is to red. The number of differentially expressed unigenes contained in each pathway is represented by the size of the dot

Genes control phenotypes, and differential expression of genes makes phenotypes different among individuals [21]. We compared the transcriptome data with the anthocyanin-containing lines as the experimental group (Anth) and the anthocyanin-free lines as the control group (CK), and found that 2329 unigenes were significantly differentially expressed between these two groups. Among them, 1235 unigenes were up-regulated and 1094 unigenes were down-regulated (Additional file 5: Figure S4). GO enrichment analysis found that the differentially expressed unigenes were significantly enriched in molecular function and biological process. Within the molecular function category, the majority of the GO terms were assigned to catalytic activity (735 unigenes) and oxidoreductase activity (194 unigenes). For biological process category, the assignments were single-organism metabolic process (314 unigenes), oxidation-reduction process (183 unigenes) and carbohydrate metabolic process (104 unigenes) (Additional file 6: Figure S5). KEGG enrichment analysis found that the up-regulated unigenes were significantly enriched in the flavonoid biosynthesis (46 unigenes), phenylpropanoid biosynthesis pathways (33 unigenes), and glutathione metabolism (31 unigenes) (Fig. 3), and the down-regulated unigenes were significantly enriched in the plant hormone signal transduction pathway (24 unigenes) (Additional file 7: Figure S6).

### Differentially expressed unigenes related to anthocyanin biosynthesis

**Fig. 4:**
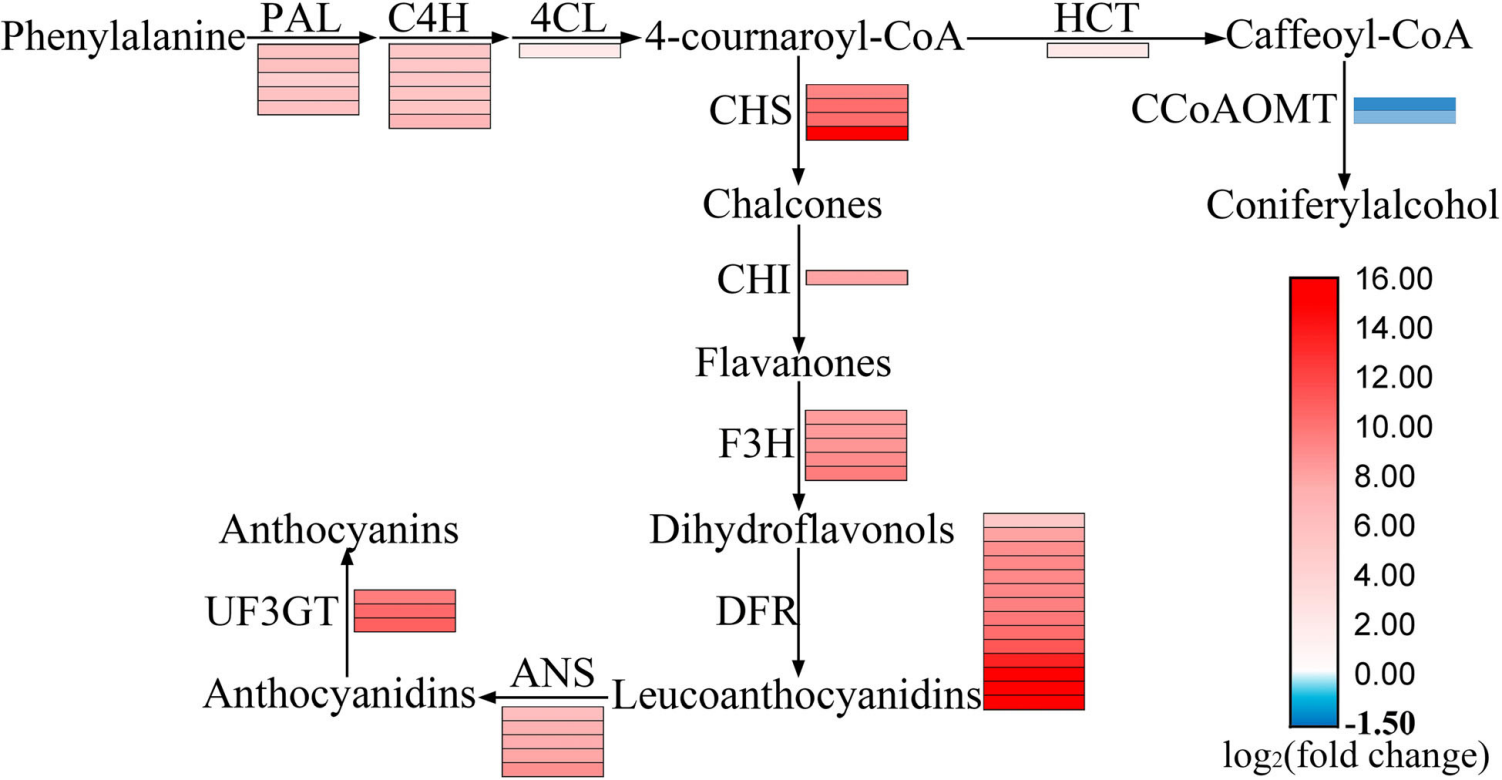
Putative biosynthetic pathways of anthocyanin in sweet potato and expression patterns of anthocyanin biosynthetic genes. Each grid represents a unigene

We identified 54 unigenes enriched in flavonoid biosynthesis pathways and 81 unigenes enriched in phenylpropanoid biosynthesis pathways from the transcriptome data. Analysis of differentially expressed unigenes between these two groups (anthocyanin-containing lines and anthocyanin-free lines) revealed that 49 unigenes were differentially expressed in the flavonoid biosynthesis pathway, of which 46 were up-regulated and 3 were down-regulated. There were 38 unigenes differentially expressed in the phenylpropanoid biosynthesis pathway, of which 33 were up-regulated and 5 were down-regulated (Additional file 8: Table S2). Analysis of differentially expressed unigenes revealed that 54 unigenes, which was up-regulated expression (log_2_ (fold change) > 2, qvalue < 0.01), were involved in anthocyanin biosynthesis. These genes include phenylalanine ammonialyase (*PAL*), trans-cinnamate 4-monooxygenase (*C4H*), 4-Coumarate:CoA ligase (*4CL*), chalcone synthase (*CHS*), chalcone-flavanone isomerase (*CHIL*), flavanone 3-hydroxylase (*F3H*), dihydroflavonol-4-reductase (*DFR*), leucoanthocyanidin dioxygenase (*LDOX/ANS*), anthocyanidin 3-O-glucoside 2-O-xylosyltransferase (*UF3GT*), UDP-glucosyltransferase 78D2 (*UGT78D2*) (Fig. 4 and Additional file 8: Table S2). These included most of genes, involved in the biosynthesize anthocyanin from phenylalanine.

### Differentially expressed transcription factors

Transcription factors (TFs) are trans-acting factors that can bind to genes promoter region, regulate genes temporal and spatial expression [22]. We used the iTAK software to predict plant transcription factors [23]. A total of 29 types of 828 transcription factors were found (Additional file 9: Table S3). Among them, the three largest types were C3H (63 unigenes), bZIP (55 unigenes) and bHLH (53 unigenes). bHLH transcriptome factors are involved in the regulation of anthocyanin biosynthesis [8]. In addition, 20 MYB transcription factors, which may be involved in anthocyanin biosynthesis, were found. The expression level of transcription factor genes determines the expression level of their control genes. Through comparison between these two groups (Anth and CK), a total of 165 transcription factor genes were found to be differentially expressed, of which, 55 were significantly up-regulated and 110 were significantly down-regulated (Additional file 10: Figure S7 and Additional file 11: Table S4).

### Differentially expressed unigenes in hormone signal transduction pathways

Plant hormones regulate plant growth, development and secondary biomass synthesis [14]. Through comparison between two groups (Anth and CK), a total of 33 hormone signal transduction pathway unigenes were found to be differentially expressed, of which 9 were significantly up-regulated and 24 were significantly down-regulated (Additional file 12: Table S5). The down-regulated unigenes were significantly enriched in signal transduction pathways such as auxin, cytokinin, brassinolide, abscisic acid (ABA), gibberellic acid (GA), and jasmonic acid (JA) (Fig. 3b). The up-regulated genes were not significantly enriched by KEGG analysis.

### Differentially expressed lncRNAs

Plant long noncoding RNAs (lncRNAs) are involved in the regulation of biological processes, including plant development, response to stresses, and secondary metabolism [24]. A total of 2054 lncRNAs were predicted by CPC, CNCI, plek and pfam software, and their lengths were from 200 to 3356 bp. Through comparison between two groups (Anth and CK), a total of 228 were found to be differentially expressed, of which 110 were significantly up-regulated and 118 were significantly down-regulated (Additional file 13: Table S6).

### Weighted gene co-expression network analysis (WGCNA) of differentially expressed unigenes

**Fig. 5:**
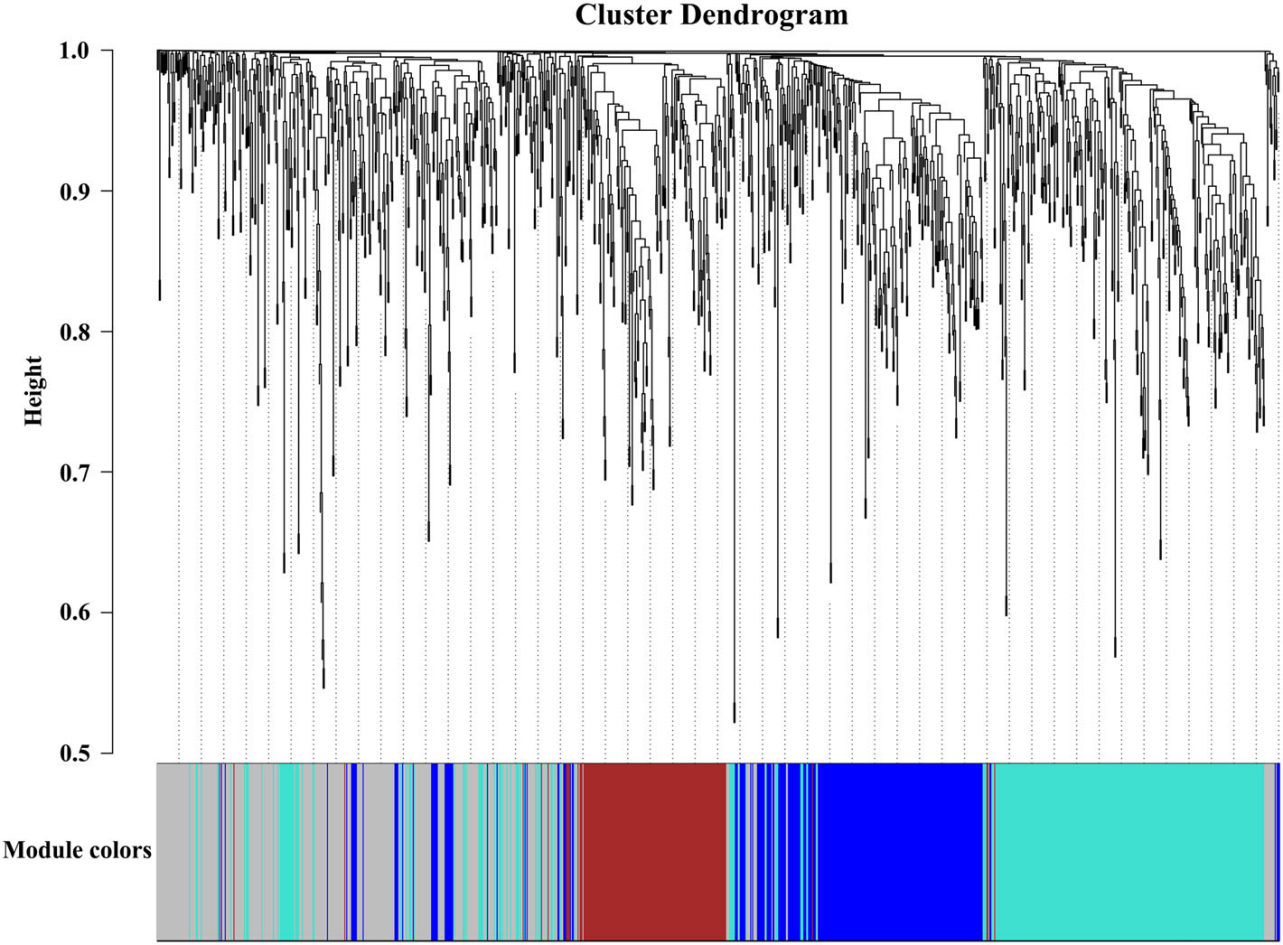
Cluster dendrogram of DEGs based on WGCNA analysis

**Fig. 6:**
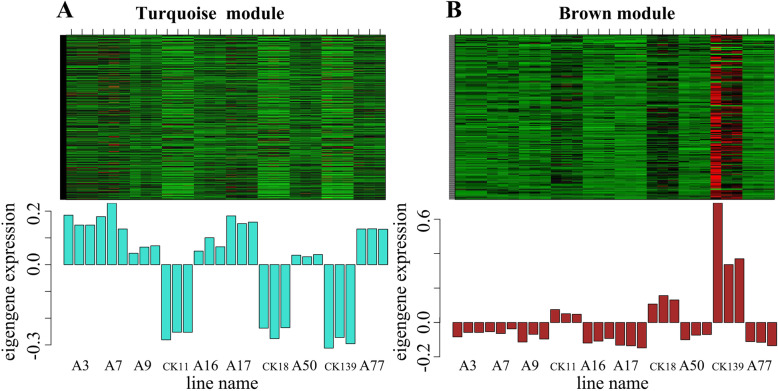
Co-analysis diagram of modules and traits. A. Co-analysis diagram of the turquoise module and traits. B. Co-analysis diagram of the brown module and traits

Genes with similar functions have similar expression patterns. WGCNA can be used to discover new genes involved in a given process [25]. Based on this, we performed a WGCNA of undirected networks on the differentially expressed unigene between two groups (Anth and CK). The clustering analysis of the samples showed that the anthocyanin-containing group (Anth) and the anthocyanin-free group (CK) were clearly divided into two categories (Additional file 14: Figure S8). The differentially expressed unigenes were aggregated into 4 modules, which are represented by turquoise, grey, brown and blue, respectively (Fig. 5). Correlation analysis between modules and traits showed that the turquoise module was significantly associatied with anthocyanin-containing group (Anth) (Fig. 6a and Additional file 15: Figure S9) and the brown module was significant association with anthocyanin-free group (CK) (Fig. 6b and Additional file 15: Figure S9). In the turquoise module, GO enrichment analysis showed that the unigenes were mainly involved in catalytic activity and oxidoreductase activity in molecular function category and oxidation-reduction process in biological process category (Additional file 16: Figure S10). KEGG enrichment analysis showed that the unigenes were significantly enriched in flavonoid biosynthesis, phenylalanine metabolism, phenylpropanoid biosynthesis and glutathione metabolism (Additional file 17: Figure S11). In the brown module, GO and KEGG enrichment analysis did not find significant gene enrichment (Additionalfile 18: Figure S12).

### Verification of differentially expressed unigenes by qRT-PCR

Based on the transcriptome data, we randomly selected twelve unigenes for qRT-PCR verification in anthocyanin-containing lines and anthocyanin-free lines. The qRT-PCR primers are listed in Additional file 19: Table S7. The qRT-PCR results and FPKM of the twelve unigenes are shown in the Additional file 20: Figure S13, which was consistent with those from the transcriptome data (r^2^ = 0.9060), indicating that the transcriptome data were reliable.

## Discussion

Sweet potato is a highly heterozygous hexaploid (2n = 6x = 90) crop with a complex genetic background. Under these conditions, to investigate the mechanism of anthocyanin biosynthesis in sweet potato storage roots by transcriptome analysis, anthocyanin-related differentially expressed genes can be masked by background differentially expressed genes. In this study, we selected Ningzishu 1 and Jizishu 2 as the parents for sexual crosses, and selected seven anthocyanin-containing lines and three anthocyanin-free lines in the F_1_ hybrid population as experimental materials. The lines were analyzed for full-length transcriptome and then compared between these two groups (Anth and CK) by the DEseq method with qvalue < 0.05. This could effectively remove the background differentially expressed genes between the experimental group (Anth) and the control group (CK), so as to find the true anthocyanin-related differentially expressed genes. In plants, anthocyanins are biosynthesized through the flavonoid biosynthesis pathway, and the upstream pathway is phenylpropanoid biosynthesis. KEGG analysis showed that the up-regulated differentially expressed unigenes were significantly enriched in these two biosynthesis pathways (Fig. 3a), which were also the two most abundant signal pathways for enrichment. This showed that our experimental results could reveal the mechanism of anthocyanin biosynthesis in sweet potato storage roots.

Compared with second-generation sequencing, full-length transcriptome sequencing is more accurate in identifying homologous genes. We performed a third-generation full-length transcriptome sequencing using PacBio sequel platform with single-molecule real-time, and 18,176 high-quality unigenes were obtained. So far, there were three transcriptome articles about the anthocyanin biosynthesis of sweet potato storage roots. Xie [15] compared two RNA-sequence datasets of Jingshu 6 (27.02 mg/100 g FW) and Guangshu 87, which is a yellow sweet potato, and found that UDP-glucose-flavonoid 3-*O*-glucosyltransferase is one of the key enzymes in anthocyanin biosynthesis. Ma [16] and Zhao [17] discovered many structural genes and transcription factors related to anthocyanin biosynthesis through transcriptome analysis of sweet potato mutants. The sweet potato used by Ma is Ningzishu 1 (22.41 mg/100 g FW) and its white flesh mutant. The sweet potato used by Zhao is Jingshu 6 (27.02 mg/100 g FW) and its mutant JS6–5 (79.80 mg/100 g FW) with high anthocyanin content. In this study, we selected anthocyanin-containing lines and anthocyanin-free lines in the F_1_ population of Ningzishu 1 and Jizishu 2 for comparative transcriptome analysis and found that anthocyanin biosynthesis genes were up-regulated expression. Five and six unigenes annotated to PAL and C4H, respectively, which participate in the transformation process from phenylalanine to 4-cournaroyl-CoA, showed an up-regulate expression. The LDOX/ANS is a 2-oxoglutarate-dependent oxygenase enzyme that converts leucoanthocyanidins to anthocyanidins, an essential step in the formation of colored metabolites in anthocyanin biosynthesis [26]. In this study, we found that five unigenes annotated with LDOX/ANS were up-regulated. The UF3GT, as the major control point for anthocyanin production, transfers glucose to the C-3 hydroxyl group of anthocyanidins [27], leading to the production of colored pigments of anthocyanins 3-O-glucosides [28]. It is the key step for anthocyanin stability and water solubility in plants [29]. In this study, we found that three unigenes annotated with UG3GT were up-regulated. Comparing our result with that from the Ningzishu 1 mutant [16], we found more homologous unigenes for anthocyanin biosynthesis. We speculated the full-length transcriptome sequencing could identify more homologous genes sequences than the second-generation sequencing.

Anthocyanin biosynthesis in plants is governed by a regulatory network that consists of the MYB-bHLH-WD40 ternary complex [30]. Regulatory genes include *MYB75* (*IbMYB1*, transcript18862), *MYB114* (transcript13511), and *bHLH2*/*TT8* (transcript2642). *AtMYB75*, as a transcriptional activator, promotes the biosynthesis of anthocyanins and procyanidins by regulating the expression of *CHS*, *DFR* and *LDOX* [31, 32]. *IbMYB1* controls anthocyanin biosynthesis in the flesh of sweet potato storage roots [33]. *AtMYB114*, as a MYB transcriptional activator, affects the expression of enzymes involved in later steps of anthocyanin biosynthesis [34]. *AtTT8*, as a helix-loop-helix (bHLH2) transcription activator, is involved in the control of flavonoid pigmentation by regulating dihydroflavonol-4-reductase (*DFR*) [34]. These unigenes, which encode the above genes, were up-regulated expression in the anthocyanin-containing lines. In this study, we found twelve differentially expressed unigenes encoding eight *MYB* genes and seventeen differentially expressed unigenes encoding nine *bHLH* genes. Fewer *MYB* and *bHLH* genes involved in anthocyanin biosynthesis were identified compared with those identified in Ningzishu 1 mutant [16]. We speculated that multiple lines comparison in the population could effectively remove background differentially expressed genes. These results showed that our experiments could more accurately reflect the mechanism of anthocyanin biosynthesis in sweet potato storage roots.

In addition, some members of the WRKY [9], MADs [35], NAC [11], AP2/ERF [36], and bZIP [10] families have been reported to be involved in the regulation of anthocyanin biosynthesis. In this study, we found that 55 transcription factors were up-regulated and 110 transcription factors were down-regulated between anthocyanin-containing group (Anth) and anthocyanin-free group (CK) (Additional file 11: Table S4). WGCNA analysis found there were a total of 10 up-regulated unigenes, encoding 7 transcription factors, and 9 down-regulated unigenes, encoding 9 transcription factors in the turquoise module (Additional file 11: Table S4). There were 2 unigenes (transcript7517 and 11,522), encoding a WRKY transcription factor, which were up-regulated expression in the anthocyanin-containing lines. There were 2 unigenes (transcript9883 and transcript11323), encoding NF-YA10 transcription factors, 1 unigene (transcript31941), encoding a B-box type zinc finger protein, 1 unigene (transcript4453), encoding a GRAS family transcription factor, 1 unigene (transcript12965), encoding a Zinc finger, and 1 unigene (transcript22607), encoding a MADS-box protein, which were down-regulated expression in the anthocyanin-containing lines. In the brown module, there were a total of 12 down-regulated unigenes, encoding 11 transcription factors and no up-regulated unigenes encoding transcription factors were found (Additional file 11: Table S4). Differential expression of transcription factors between anthocyanin-containing group and anthocyanin-free group indicated that anthocyanin biosynthesis in sweet potato storage roots was regulated at the transcription levels. Our results provided a basis for discovering new transcription factors that regulate anthocyanin biosynthesis.

To investigate the potential role of hormones in anthocyanin biosynthesis, we analyzed the expression of genes related to plant hormone signal transduction. In the GA signal transduction pathway, binding of GA to its receptor GID1 leads to DELLAs degradation via the ubiquitin-proteasome pathway [37]. The DELLA proteins promote anthocyanin biosynthesis via sequestering JAZ suppressors of the MYB/bHLH/WD40 complex and subsequent release of PAP1 and TT8 in *Arabidopsis thaliana* [38]. Transcriptome analysis showed that one unigene (transcript14390) encoding *GID1* was significantly down-regulated expression. While the expression of unigene (transcript16804) encoding *JAZ* did not change significantly. These unigenes (transcript2642 and transcript2950) encoding *TT8* were significantly up-regulated expression in the anthocyanin-containing lines. Based on these results, we speculated that by reducing the expression of the *GID1* gene in sweet potato, GA’s inhibition of anthocyanin biosynthesis was lifted and anthocyanin biosynthesis was promoted. However, the negative regulation of *JAZ* gene on anthocyanin biosynthesis in sweet potato might not be at the transcriptome level.

Studies have shown that many lncRNAs have conserved secondary structures and spliced forms [39], and are regulated in specific tissues, cells, and developmental stages [40]. Functional analyses have shown that lncRNAs regulate gene expression at the transcriptional [41], post-transcriptional [42], and epigenetic levels [43]. A total of 2054 lncRNAs were identified by full-length transcriptome analyses. Through comparisons between groups, we found 110 up-regulated lncRNAs and 118 down-regulated lncRNAs (Additional file 13: Table S6). WGCNA analyses showed 24 lncRNAs were up-regulated, and 11 lncRNAs were down-regulated in the turquoise module. Based on these results, we speculated that lncRNAs played a regulatory role in anthocyanin biosynthesis in sweet potato storage roots.

## Conclusions

A total of 18,176 sweet potato unigenes were identified by a full-length transcriptome sequencing analysis of a sweet potato F_1_ hybrid population. In our study, we identified more homologous genes involved in anthocyanin biosynthesis in sweet potato compared with previous transcriptome studies. By comparing the full-length transcriptomes of seven anthocyanin-containing lines and three anthocyanin-free lines in the F_1_ hybrid population and combining with WGCNA analysis, we found that, in addition to the MBW regulatory complexes (MYB, bHLH, and WD40), transcription factors such as WRKY, NF-YA10, GRAS, and MADS-box were also involved in the regulation of anthocyanin biosynthesis. It was also found that hormones and lncRNAs might be involved in the regulation of anthocyanin biosynthesis at the transcriptome level.

## Methods

### Plant materials

We selected the sweet potato cultivar Ningzishu 1 (22.41 mg/100 g FW), which was bred by the Jiangsu Academy of Agricultural Sciences, and cultivar Jizishu 2 (132.95 mg/100 g FW), which was bred by the Crop Research Institute of Shandong Academy of Agricultural Sciences, as the female and male parent, respectively. These two parents were treated with a short photoperiod (8 h light / 16 h dark) in a dark room to induce flowering, followed by artificial pollination and seed collection. A total of 256 lines with isolated traits were obtained.

The storage roots of seven anthocyanin-containing lines and three anthocyanin-free lines were harvested on the 90th day after transplanting the seedlings. The cylinders with a diameter of 0.5 cm from the middle part of medium-sized storage roots were collected, and quickly frozen in liquid nitrogen for transcriptome sequencing. The remainders were used to determine the anthocyanin content.

### Determination of anthocyanin

The anthocyanin content of sweet potato storage roots was measured by enzyme linked immunosorbent assay (ELISA) referring to the method of Li [44], with no less than six independent biological replicates per lines.

### RNA isolation and full cDNA library construction

The total RNAs were extracted with RNAprep Pure Plant Plus Kit (Polysaccharides & Polyphenolics-rich) (Tiangen Biotech (Beijing) CO. LTD.). The extracted total RNA was subjected to agarose gel electrophoresis to analyze the RNA degradation and contamination degree. RNA purity was analyzed by Nanodrop. RNA concentration was measured by Qubit. RNA integrity was analyzed by Agilent 2100.

The full-length cDNA library construction was as follows: (1) Use oligo (dT) to enrich mRNA containing polyA; (2) Reverse transcription of mRNA into cDNA using a SMARTer PCR cDNA Synthesis Kit; (3) PCR amplification of enriched cDNA; (4) Use magnetic beads to select fragments for large-scale PCR to obtain sufficient total cDNAs; (5) Full-length cDNA for damage repair, end repair, and connect SMRT dumbbell-shaped adapters to construct a full-length transcriptome library; (6) Digestion with exonuclease to remove the unlinked linker sequences at both ends of the cDNA; (7) Finally, primers and DNA polymerase were bound to form a complete SMRT bell library. RNA isolation and library construction of second-generation transcriptome were performed according to the method described by Qin [45].

### Sequencing, identification of DEGs and annotation

Qualified full cDNA library was sequenced using the PacBio Sequel platform in one cell. The second-generation libraries were sequenced using the Hiseq 2000 sequencing platform (Illumina) with PE150. Full cDNA sequences were processed using the SMRTlink 7.0 software and corrected by second-generation transcription data with the LoRDEC software to obtain consensus unigenes. Any redundancy unigenes were removed by CD-HIT software. Differential expression genes analysis between anthocyanin-containing lines and anthocyanin-free lines was performed using the DESeq R package (1.10.1) with qvalue < 0.05. For the anthocyanins biosynthesis genes, the criterion we chose was that log_2_ (fold change) > 2, and qvalue < 0.01. Annotation of the unigenes was performed by searching against the NCBI nucleotide sequences (Nt), NCBI non-redundant protein (NR), Gene Ontology (GO), Kyoto Encyclopedia of Genes and Genomes (KEGG), euKaryotic Ortholog Groups (KOG), Protein family (Pfam) and Swiss-Prot databases with e-value = 1e-5.

### LncRNA prediction

We used PLEK [46], CNCI [47], CPC2 [48], and Pfam [49] to predict unigenes coding potential. First, both PLEK and CNCI software were used to predict the coding potential based on the sequence characteristics of the unigenes. Second, the CPC2 software was used to evaluate the coding potential of the unigenes based on the biological sequence characteristics of each coding frame of the transcript. Third, the unigenes sequence predicted by the above software was subjected to homology search in the Pfam database by hmmscan, and finally the lncRNA sequence was obtained.

### qRT-PCR validation of the transcriptome data

Twelve unigenes were selected to examine their expression in anthocyanin-containing lines and anthocyanin-free lines. The qRT-PCR primer pairs of unigenes were designed using primer 5. qRT-PCR was performed using a Roche LightCycler® 480II system with the TaKaRa SYBR Premix Ex Taq™ (Tli RNaseH Plus) under the following conditions: 95 °C for 10 s, followed by 40 cycles of 95 °C for 15 s, 55 °C for 15 s and 72 °C for 15 s. The expression levels of genes were normalized to the level of constitutive *Ibactin* expression. The qRT-PCR results were analyzed using the 2^−△△CT^ method [50].

## Supplementary information

**Additional file 1: Table S1.** Samples person analysis.

**Additional file 2: Figure S1.** GO classification of unigenes.

**Additional file 3: Figure S2.** KOG classification of unigenes.

**Additional file 4: Figure S3.** KEGG classification of unigenes.

**Additional file 5: Figure S4.** Volcano plot of differentially expressed unigenes.

**Additional file 6: Figure S5.** Enriched GO terms of differentially expressed unigenes.

**Additional file 7: Figure S6.** KEGG enrichment analysis of down-regulated DEGs.

**Additional file 8: Table S2.** DEGs related to anthocyanin biosynthesis.

**Additional file 9: Table S3.** Transcription factor statistics.

**Additional file 10: Figure S7.** Differentially expressed transcription factor.

**Additional file 11: Table S4.** Summaries of the differentially expressed transcription factors.

**Additional file 12: Table S5.** Summaries of the differentially expressed hormone unigenes.

**Additional file 13: Table S6.** Summaries of the differentially expressed lncRNAs.

**Additional file 14: Figure S8.** WGCNA sample clustering.

**Additional file 15: Figure S9.** Modules samples relationship.

**Additional file 16: Figure S10.** The most enriched GO terms of the turquoise module.

**Additional file 17: Figure S11.** KEGG enrichment analysis of the turquoise module.

**Additional file 18: Figure S12.** KEGG enrichment analysis of the brown module.

**Additional file 19: Table S7.** The primer of qRT-PCR.

**Additional file 20: Figure S13.** qRT-PCR analysis of differentially expressed genes.

## Data Availability

All data generated in this study are included in the paper and in the supporting information files. Transcriptome data has been submitted to the NCBI SRA (PRJNA608140), https://www.ncbi.nlm.nih.gov/sra/?term=PRJNA608140. Ningzishu 1 is kept by the breeding institute Jiangsu Academy of Agricultural Sciences. Jizishu 2 and the lines used for transcriptome sequencing are kept by the breeding institute Crop Research Institute of Shandong Academy of Agricultural Sciences where the corresponding author is located.

## Abbreviations

CCS: Circular consensus sequencing
FLNC: Full-length non-chimeric
NFL: Non-full-length non-chimeric
SMRT: Single-molecule real-time
Nt: Nucleotide sequences
NR: NCBI non-redundant protein
GO: Gene Ontology
KEGG: Kyoto Encyclopedia of Genes and Genomes
KOG: Eukaryotic Ortholog Groups
Pfam: Protein family
ELISA: Enzyme linked immunosorbent assay
CK: The control group
Anth: The experimental group
FW: Fresh weight
TFs: Transcription factors
ABA: Abscisic acid
GA: Gibberellic acid
JA: Jasmonic acid
lncRNAs: Long noncoding RNAs
WGCNA: Weighted gene co-expression network analysis
PAL: Phenylalanine ammonialyase
C4H: Trans-cinnamate 4-monooxygenase
4CL: 4-Coumarate:CoA ligase
CHS: Chalcone synthase
CHIL: Chalcone-flavanone isomerase
F3H: Flavanone 3-hydroxylase
DFR: Dihydroflavonol-4-reductase
LDOX/ANS: Leucoanthocyanidin dioxygenase
UF3GT: Anthocyanidin 3-O-glucoside 2-O-xylosyltransferase
UGT78D2: UDP-glucosyltransferase 78D2
