# Supplementary figures and images for: Comparative analysis of full-length transcriptomes based on hybrid population reveals regulatory mechanisms of anthocyanin biosynthesis in sweet potato (Ipomoea batatas (L.) Lam)
Source: BMC Plant Biol. 2020 Jun 29;20:299. doi: 10.1186/s12870-020-02513-1 (PMC7325064; doi:10.1186/s12870-020-02513-1)

# Gene Function Classification (GO)

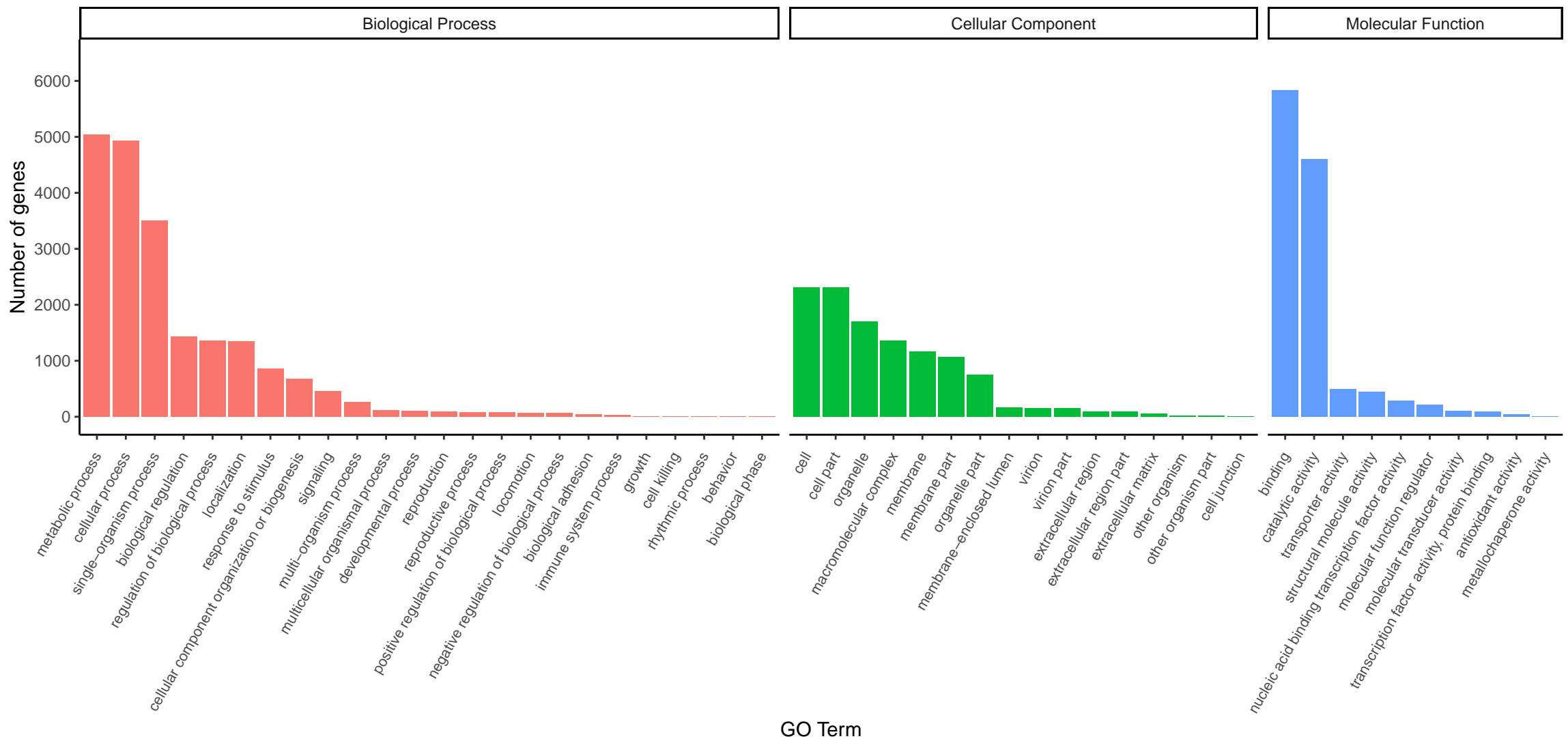

Supplement: Supplementary file 2 — Additional file 2: Figure S1. GO classification of unigenes. [file 12870_2020_2513_MOESM2_ESM.pdf]

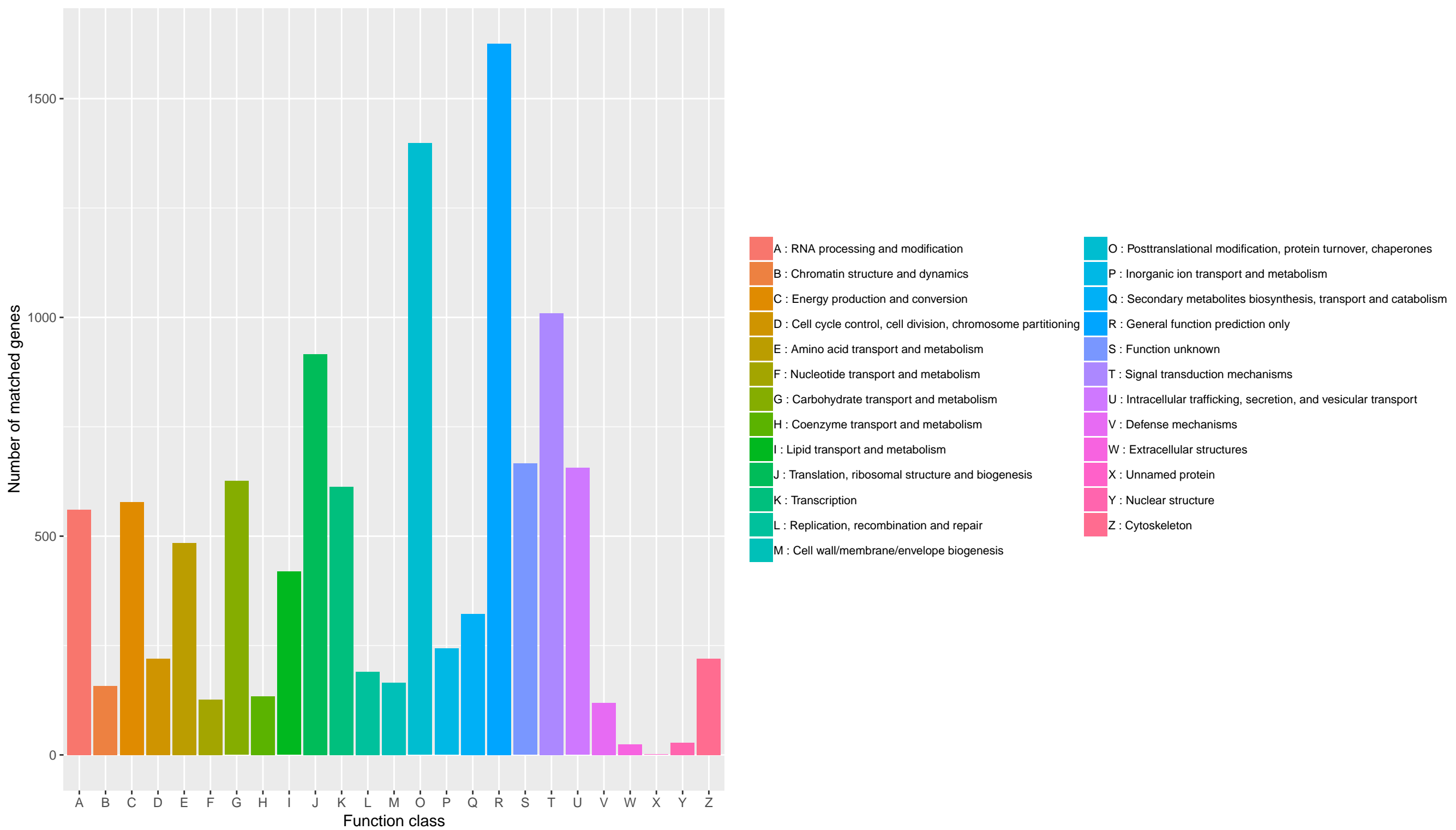

Supplement: Supplementary file 3 — Additional file 3: Figure S2. KOG classification of unigenes. [file 12870_2020_2513_MOESM3_ESM.pdf]

# Anth vs CK

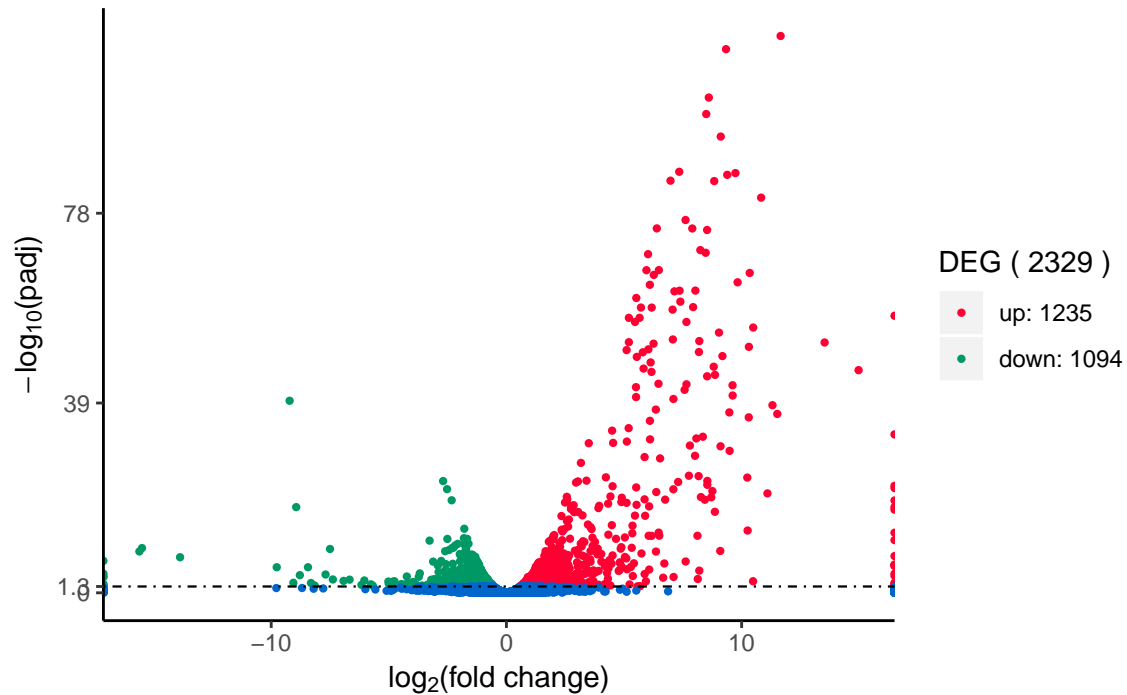

Supplement: Supplementary file 5 — Additional file 5: Figure S4. Volcano plot of differentially expressed unigenes. [file 12870_2020_2513_MOESM5_ESM.pdf]

## Differentially expressed transcription factor (Anth VS CK)

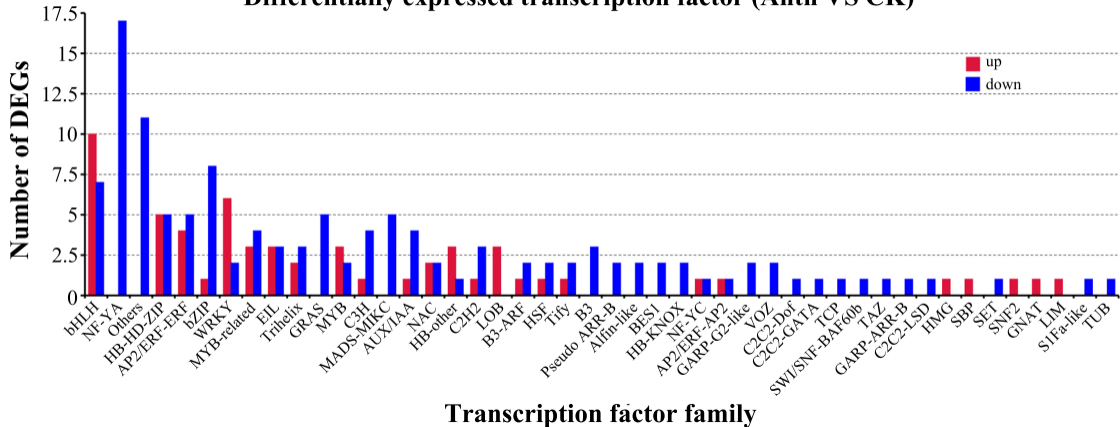

Supplement: Supplementary file 10 — Additional file 10: Figure S7. Differentially expressed transcription factor. [file 12870_2020_2513_MOESM10_ESM.pdf]

Sample clustering to detect outliers

Height

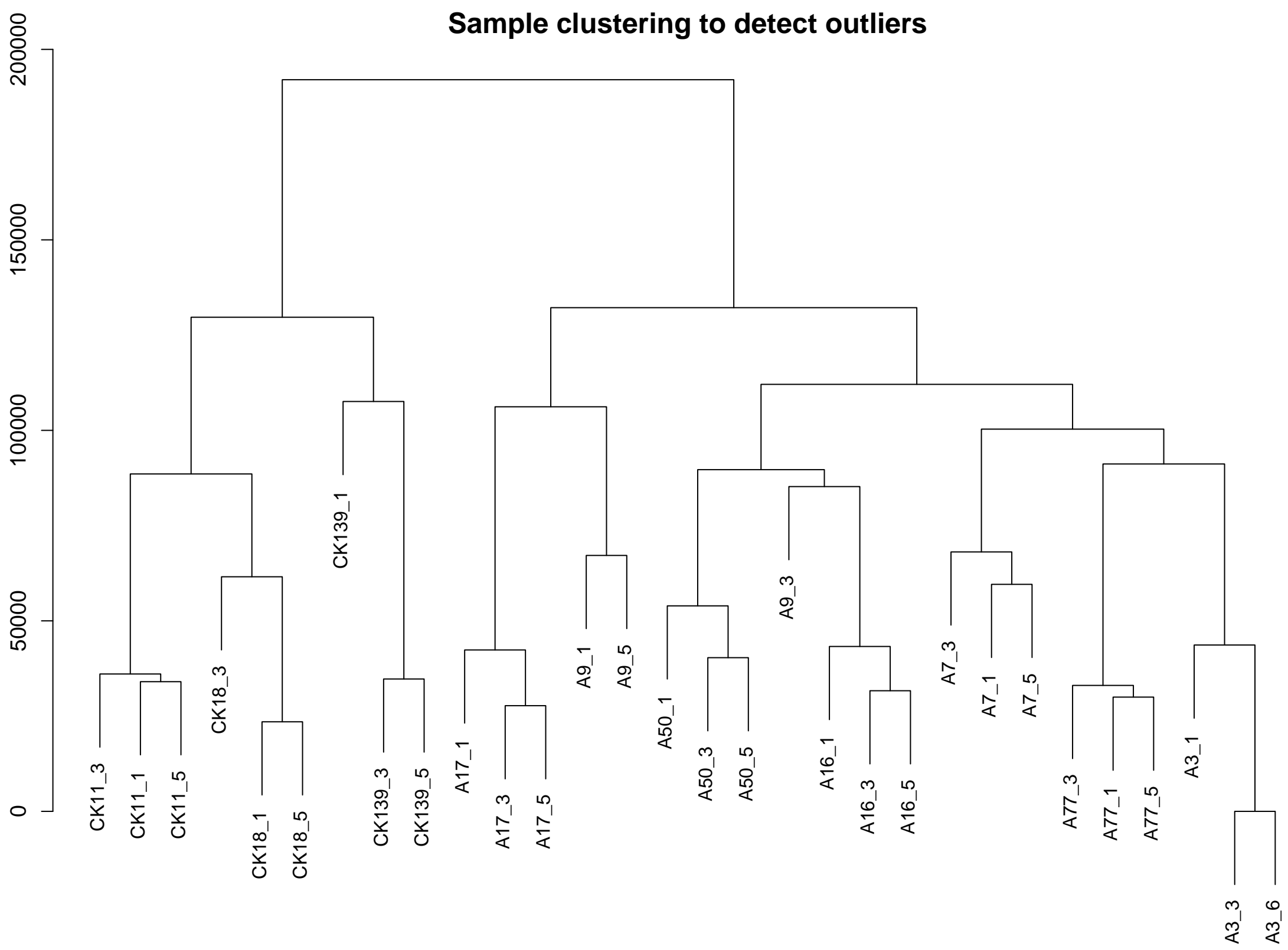

Supplement: Supplementary file 14 — Additional file 14: Figure S8. WGCNA sample clustering. [file 12870_2020_2513_MOESM14_ESM.pdf]

# Module-Sample relationship

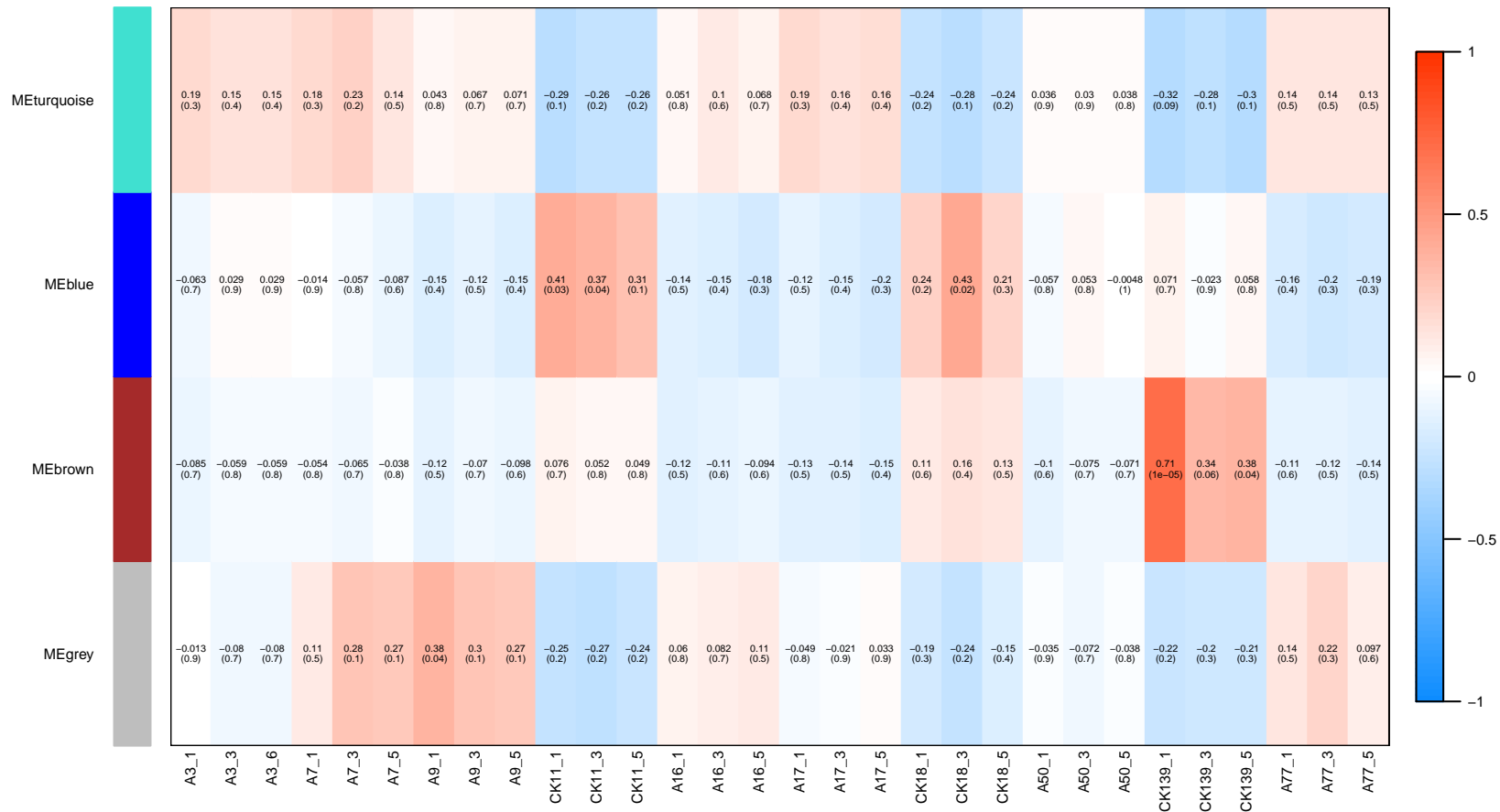

Supplement: Supplementary file 15 — Additional file 15: Figure S9. Modules samples relationship. [file 12870_2020_2513_MOESM15_ESM.pdf]

# The Most Enriched GO Terms

GO term

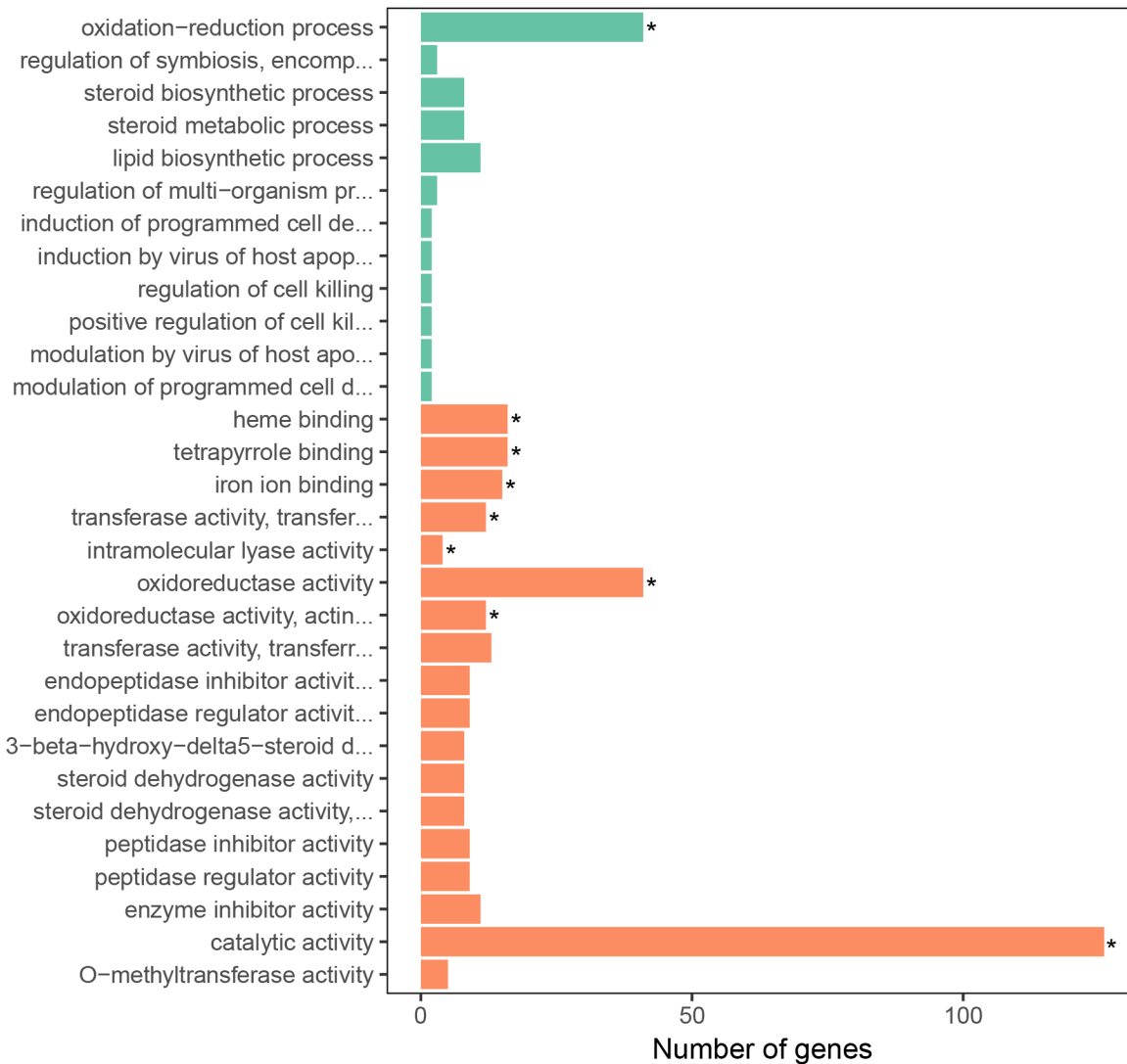

type

biological\_process  
molecular\_function

Supplement: Supplementary file 16 — Additional file 16: Figure S10. The most enriched GO terms of the turquoise module. [file 12870_2020_2513_MOESM16_ESM.pdf]

# Statistics of Pathway Enrichment

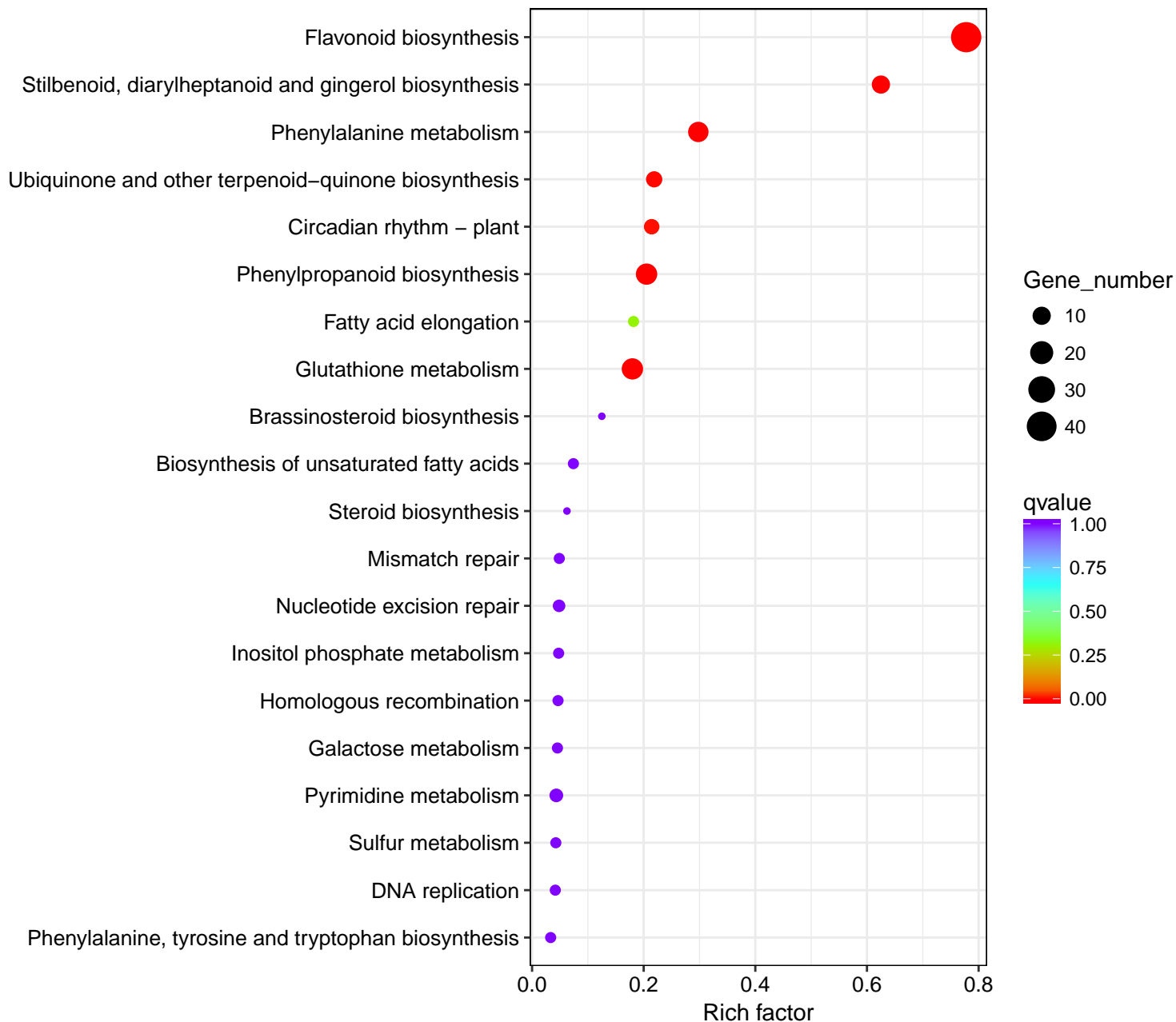

Supplement: Supplementary file 17 — Additional file 17: Figure S11. KEGG enrichment analysis of the turquoise module. [file 12870_2020_2513_MOESM17_ESM.pdf]

# Statistics of Pathway Enrichment

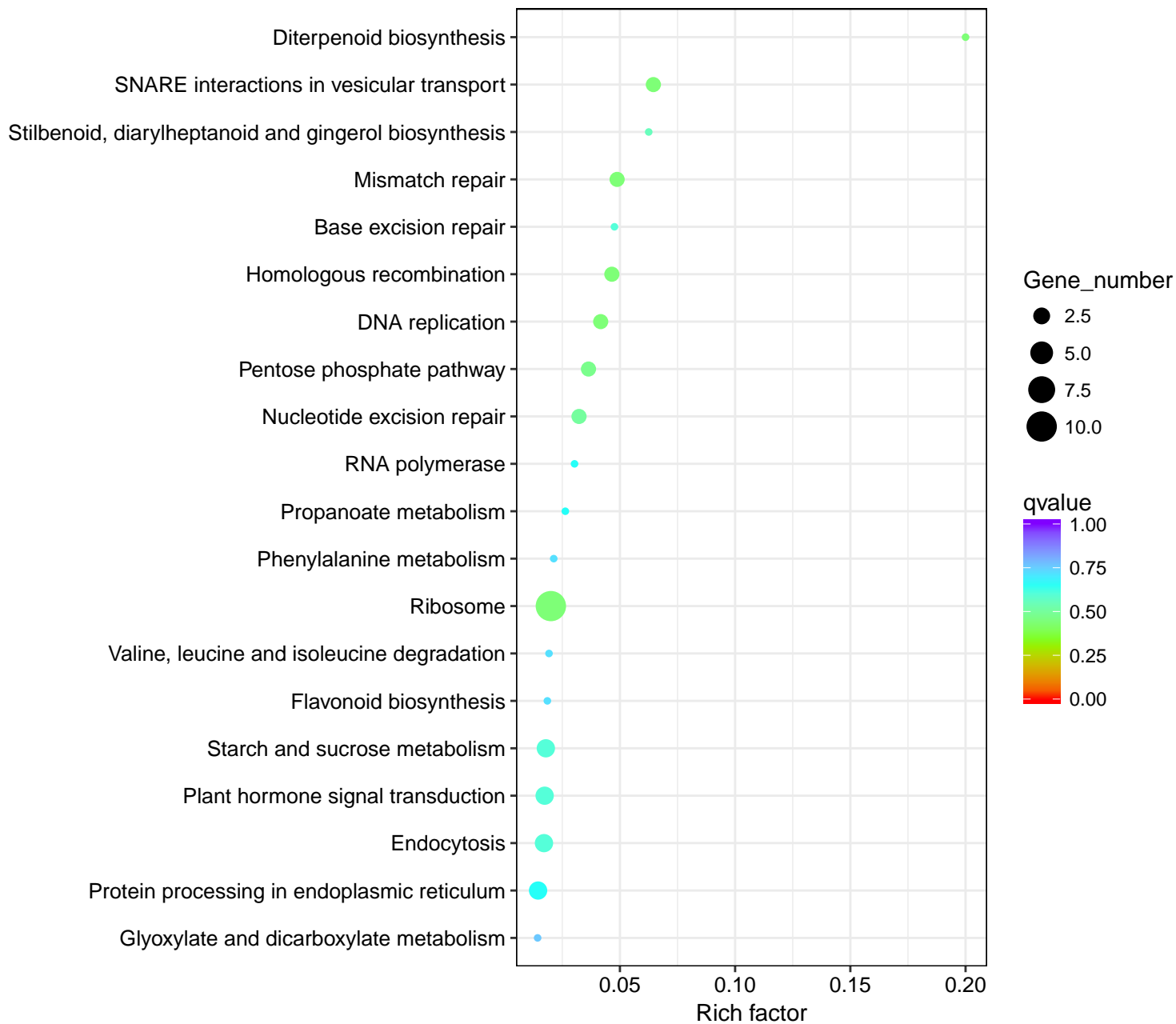

Supplement: Supplementary file 18 — Additional file 18: Figure S12. KEGG enrichment analysis of the brown module. [file 12870_2020_2513_MOESM18_ESM.pdf]

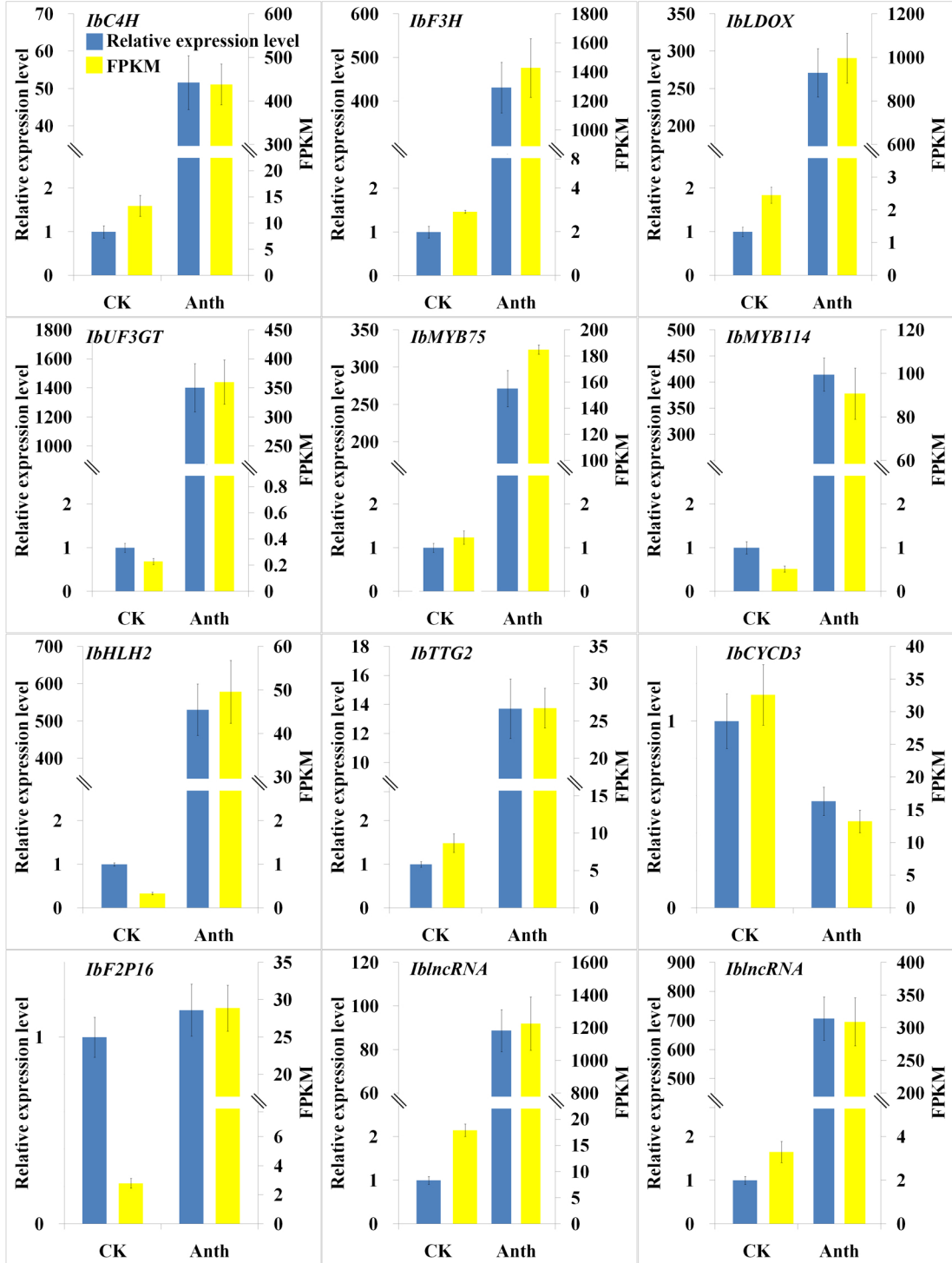

Supplement: Supplementary file 20 — Additional file 20: Figure S13. qRT-PCR analysis of differentially expressed genes. [file 12870_2020_2513_MOESM20_ESM.pdf]
