# Supplementary material for: Comparative analysis of full-length transcriptomes based on hybrid population reveals regulatory mechanisms of anthocyanin biosynthesis in sweet potato (Ipomoea batatas (L.) Lam)
Source: BMC Plant Biol. 2020 Jun 29;20:299. doi: 10.1186/s12870-020-02513-1 (PMC7325064; doi:10.1186/s12870-020-02513-1)
Supplement: Supplementary file 4 — Additional file 4: Figure S3. KEGG classification of unigenes. [file 12870_2020_2513_MOESM4_ESM.pdf]

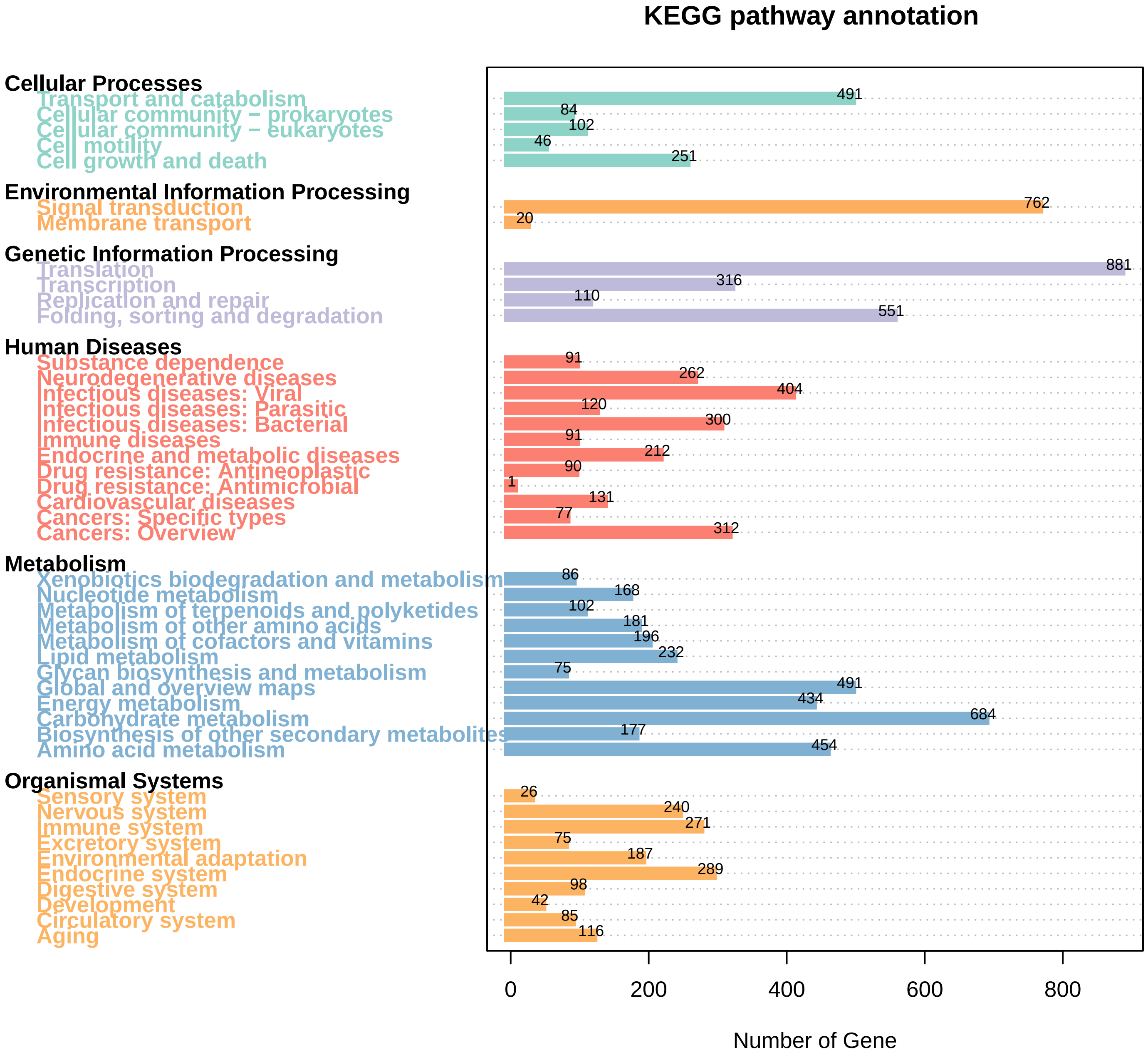
KEGG pathway annotation
Cellular Processes
Transport and catabolism
Cellular community − prokaryotes
Cellular community − eukaryotes
Cell motility
Cell growth and death
Environmental Information Processing
Signal transduction
Membrane transport
Genetic Information Processing
Translation
Transcription
Replication and repair
Folding, sorting and degradation
Human Diseases
Substance dependence
Neurodegenerative diseases
Infectious diseases: Viral
Infectious diseases: Parasitic
Infectious diseases: Bacterial
Immune diseases
Endocrine and metabolic diseases
Drug resistance: Antineoplastic
Drug resistance: Antimicrobial
Cardiovascular diseases
Cancers: Specific types
Cancers: Overview
Metabolism
Xenobiotics biodegradation and metabolism
Nucleotide metabolism
Metabolism of terpenoids and polyketides
Metabolism of other amino acids
Metabolism of cofactors and vitamins
Lipid metabolism
Glycan biosynthesis and metabolism
Global and overview maps
Energy metabolism
Carbohydrate metabolism
Biosynthesis of other secondary metabolites
Amino acid metabolism
Organismal Systems
Sensory system
Nervous system
Immune system
Excretory system
Environmental adaptation
Endocrine system
Digestive system
Development
Circulatory system
Aging
491
84
102
46
251
762
20
881
316
110
551
91
262
404
120
300
91
212
90
1
131
77
312
86
168
102
181
196
232
75
491
434
684
177
454
26
240
271
75
187
289
98
42
85
116
0
200
400
600
800
Number of Gene
