# Supplementary material for: Comparative analysis of full-length transcriptomes based on hybrid population reveals regulatory mechanisms of anthocyanin biosynthesis in sweet potato (Ipomoea batatas (L.) Lam)
Source: BMC Plant Biol. 2020 Jun 29;20:299. doi: 10.1186/s12870-020-02513-1 (PMC7325064; doi:10.1186/s12870-020-02513-1)
Supplement: Supplementary file 7 — Additional file 7: Figure S6. KEGG enrichment analysis of down-regulated DEGs. [file 12870_2020_2513_MOESM7_ESM.pdf]

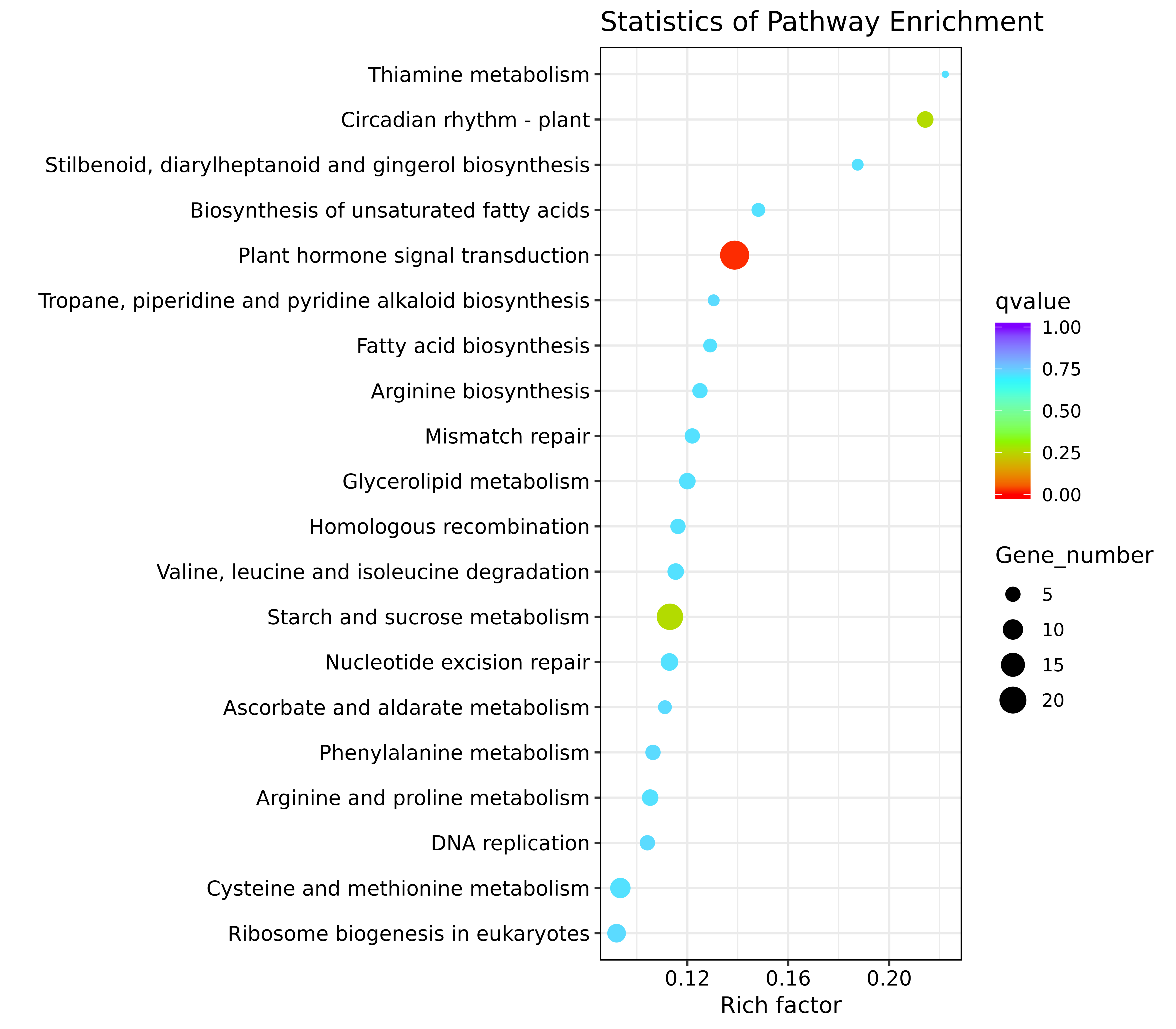
Statistics of Pathway Enrichment
Thiamine metabolism
Circadian rhythm - plant
Stilbenoid, diarylheptanoid and gingerol biosynthesis
Biosynthesis of unsaturated fatty acids
Plant hormone signal transduction
Tropane, piperidine and pyridine alkaloid biosynthesis
Fatty acid biosynthesis
Arginine biosynthesis
Mismatch repair
Glycerolipid metabolism
Homologous recombination
Valine, leucine and isoleucine degradation
Starch and sucrose metabolism
Nucleotide excision repair
Ascorbate and aldarate metabolism
Phenylalanine metabolism
Arginine and proline metabolism
DNA replication
Cysteine and methionine metabolism
Ribosome biogenesis in eukaryotes
0.12
0.16
0.20
Rich factor
qvalue
1.00
0.75
0.50
0.25
0.00
Gene_number
5
10
15
20
