# Supplementary material for: Comparative analysis of full-length transcriptomes based on hybrid population reveals regulatory mechanisms of anthocyanin biosynthesis in sweet potato (Ipomoea batatas (L.) Lam)
Source: BMC Plant Biol. 2020 Jun 29;20:299. doi: 10.1186/s12870-020-02513-1 (PMC7325064; doi:10.1186/s12870-020-02513-1)
Supplement: Supplementary file 6 — Additional file 6: Figure S5. Enriched GO terms of differentially expressed unigenes. [file 12870_2020_2513_MOESM6_ESM.pdf]

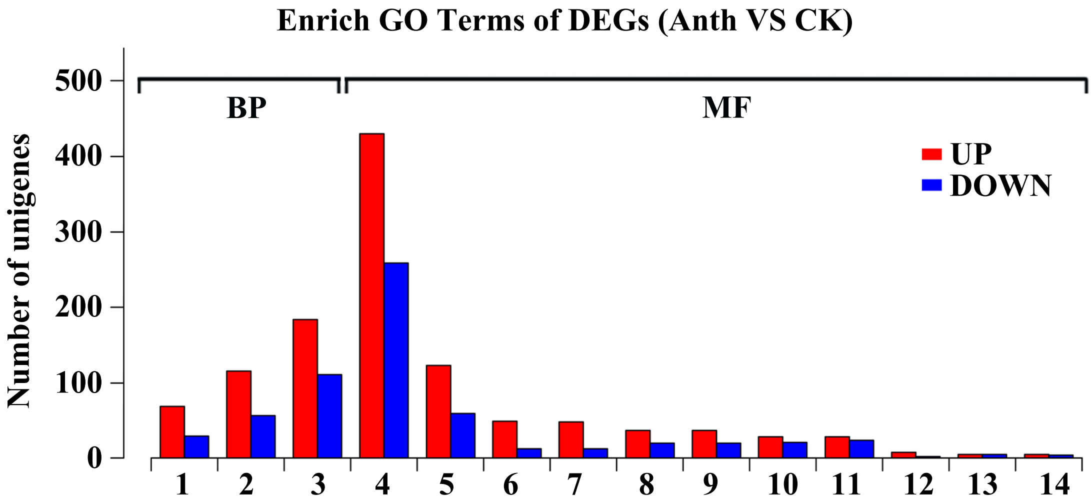

1. carbohydrate metabolic process, 2. oxidation-reduction process, 3. single-organism metabolic process, 4. catalytic activity, 5. oxidoreductase activity, 6. hydrolase activity, acting on glycosyl bonds, 7. hydrolase activity, hydrolyzing O-glycosyl compounds, 8. heme binding, 9. tetrapyrrole binding, 10. oxidoreductase activity, acting on paired donors, with incorporation or reduction of molecular oxygen, 11. iron ion binding, 12. polygalacturonase activity, 13. galactosidase activity, 14. oxidoreductase activity, acting on the aldehyde or oxo group of donors, disulfide as acceptor
